# Hydrogen cyanamide exposure: a case series from Pavia Poison Control Centre

**DOI:** 10.1093/occmed/kqad108

**Published:** 2023-11-08

**Authors:** L Bernasconi, M Carnovale, D Lonati, V M Petrolini, A Schicchi, B Brolli, V M Negrini, C Grazioli, O Maystrova, E Buscaglia, Giulia Scaravaggi, F Crema, C A Locatelli

**Affiliations:** Toxicology Unit, Istituti Clinici Scientifici Maugeri SpA SB IRCCS, Pavia Poison Control Centre, National Toxicology Information Centre, Clinical and Experimental Lab, Pavia 27100, Italy; Postgraduate School of Pharmacology and Clinical Toxicology, University of Pavia, Pavia 27100, Italy; Toxicology Unit, Istituti Clinici Scientifici Maugeri SpA SB IRCCS, Pavia Poison Control Centre, National Toxicology Information Centre, Clinical and Experimental Lab, Pavia 27100, Italy; Postgraduate School of Pharmacology and Clinical Toxicology, University of Pavia, Pavia 27100, Italy; Toxicology Unit, Istituti Clinici Scientifici Maugeri SpA SB IRCCS, Pavia Poison Control Centre, National Toxicology Information Centre, Clinical and Experimental Lab, Pavia 27100, Italy; Toxicology Unit, Istituti Clinici Scientifici Maugeri SpA SB IRCCS, Pavia Poison Control Centre, National Toxicology Information Centre, Clinical and Experimental Lab, Pavia 27100, Italy; Toxicology Unit, Istituti Clinici Scientifici Maugeri SpA SB IRCCS, Pavia Poison Control Centre, National Toxicology Information Centre, Clinical and Experimental Lab, Pavia 27100, Italy; Experimental Medicine PhD program, University of Pavia, Pavia 27100, Italy; Toxicology Unit, Istituti Clinici Scientifici Maugeri SpA SB IRCCS, Pavia Poison Control Centre, National Toxicology Information Centre, Clinical and Experimental Lab, Pavia 27100, Italy; Postgraduate School of Pharmacology and Clinical Toxicology, University of Pavia, Pavia 27100, Italy; Toxicology Unit, Istituti Clinici Scientifici Maugeri SpA SB IRCCS, Pavia Poison Control Centre, National Toxicology Information Centre, Clinical and Experimental Lab, Pavia 27100, Italy; Postgraduate School of Pharmacology and Clinical Toxicology, University of Pavia, Pavia 27100, Italy; Toxicology Unit, Istituti Clinici Scientifici Maugeri SpA SB IRCCS, Pavia Poison Control Centre, National Toxicology Information Centre, Clinical and Experimental Lab, Pavia 27100, Italy; Postgraduate School of Pharmacology and Clinical Toxicology, University of Pavia, Pavia 27100, Italy; Toxicology Unit, Istituti Clinici Scientifici Maugeri SpA SB IRCCS, Pavia Poison Control Centre, National Toxicology Information Centre, Clinical and Experimental Lab, Pavia 27100, Italy; Toxicology Unit, Istituti Clinici Scientifici Maugeri SpA SB IRCCS, Pavia Poison Control Centre, National Toxicology Information Centre, Clinical and Experimental Lab, Pavia 27100, Italy; Toxicology Unit, Istituti Clinici Scientifici Maugeri SpA SB IRCCS, Pavia Poison Control Centre, National Toxicology Information Centre, Clinical and Experimental Lab, Pavia 27100, Italy; Department of Internal Medicine and Therapeutics, Section of Pharmacology, University of Pavia, Pavia 27100, Italy; Toxicology Unit, Istituti Clinici Scientifici Maugeri SpA SB IRCCS, Pavia Poison Control Centre, National Toxicology Information Centre, Clinical and Experimental Lab, Pavia 27100, Italy

## Abstract

**Background:**

Hydrogen cyanamide is a plant growth regulator introduced in Italy as Dormex in 2000, but recalled from the market in 2008. It’s currently not authorized in Europe. Inhalation/dermal contact may cause irritation/caustic burns, ingestion of severe organ damage and concomitant alcohol consumption disulfiram-like reaction due to aldehyde-dehydrogenase inhibition by hydrogen cyanamide.

**Aims:**

To study all exposure cases referred to our centre, evaluating temporal and geographic distribution and analysing clinical manifestations, including the ones after alcohol consumption.

**Methods:**

We retrospectively evaluated all hydrogen cyanamide exposures referred to our Poison Control Centre (January 2007–December 2021). For each case, age, sex, exposure route/year, geographical location, intent of exposure, alcohol co-ingestion, emergency department-admission Poison Severity Score, signs/symptoms and treatment were analysed.

**Results:**

Thirty subjects were included. Median case/year was 1 [1; 2]: 79% occurred after market withdrawal, 92% in Sicily. All exposures were unintentional and work related; 41% of patients also co-ingested alcohol. Mean poison severity score at emergency department admission was 1.54, more severe when ingestion occurred. The most common signs/symptoms were flushing, secondary to peripheral vasodilation (41%), hyperaemia/erythema (29%), dyspnoea (25%), nausea (20%), vomiting (12%), oedema (12%), II–III degrees burns (12%) and pharyngodynia (12%). All patients were treated symptomatically and fully recovered.

**Conclusions:**

Hydrogen cyanamide exposure can lead to severe clinical manifestations. Despite its withdrawal from the Italian market, hydrogen cyanamide is still used: through PCC’s crucial role in monitoring exposure to agricultural products efforts should be made to contrast illegal trade and increase awareness of its potential toxicity in those countries in which it’s still legal.

## Teaser

Hydrogen cyanamide is a plant growth regulator recalled from the market of many countries because of its potential toxicity. Few data describing hydrogen cyanamide toxicity exist in literature; our study describes hydrogen cyanamide exposures before and after Italian market withdrawal, focusing on clinical manifestations and route of exposure. We also describe symptoms arising from concomitant hydrogen cyanamide use and alcohol consumption due to hydrogen cyanamide ability to cause disulfiram-like reactions by inhibition of aldehyde dehydrogenase.

**Key learning points**

**What is already known about this subject:**

- Hydrogen cyanamide potential toxicity is well known: it can cause irritation/caustic burns upon inhalation/dermal contact, severe organ damage after ingestion and disulfiram-like reaction (when used together with alcohol consumption) regardless of the route of exposure.
- Despite its use isn’t authorized in Europe, Pavia Poison Control Centre was consulted to manage hydrogen cyanamide exposure, even after market withdrawal.
- It’s important to raise awareness of possible risks associated with hydrogen cyanamide exposure in those countries in which it’s still commercialized or used illegally.

**What this study adds:**

- The analyses of this Italian case series increase the knowledge on hydrogen cyanamide toxicity, adding more data to the few existing in literature.
- It demonstrates that, despite its market withdrawal, it’s still used to this day in Italy, especially in the Sicily region: it may be used illegally in other countries.
- Lastly, this study shows how while using hydrogen cyanamide, especially when obtained illegally, workers are often prone to disregard safety measures, like avoiding alcohol consumption and correctly using personal protective equipment.

**What impact this may have on practice or policy:**

- In those countries in which hydrogen cyanamide is still used, it’s important to educate workers on potential severe toxicity arising from exposure and on the importance of safety occupational measures, correct use of personal protective equipment and avoid alcohol consumption.
- In Italy, crucial efforts should be made to contrast illegal trade: in addition to hydrogen cyanamide toxicity, in this context, workers lack proper education and occupational monitoring.

## Introduction

Hydrogen cyanamide (CH_2_N_2_—CAS number 420-04-2) is a plant growth regulator used in agriculture to promote uniform budding from dormant plants: it’s transformed, by soil bacteria, to ammonium carbonate that is exploited as a source of nitrogen [1]. It’s primarily used as a fertilizer but, because of its potential toxicity, its use has been restricted by some governments [2]. Hydrogen cyanamide is sold with various trade names such as Krop-Max, BudPro and, in Italy, as Dormex. Dormex requires a national authorization to be sold and is currently not authorized in Europe [3] but is regularly used in other countries such as USA, South America, India and New Zealand [4]. Its use may pose an occupational hazard, but until now, very few studies addressed this topic.

Dormex (active ingredient: 520 g/L of hydrogen cyanamide) was introduced in Italy in 2000, but a temporary suspension of its sale was established in February 2002 after a report was made to the Italian National Institute of Health (INIH) of 28 cases of hydrogen cyanamide-related poisoning [5]. Dormex sale was authorized once again in June 2003 only after the introduction of more strict protective measures [5]. However, after a few years, Dormex was definitely recalled from the Italian market by a national decree of the 18 March 2008, issued by the general director of food safety and nutrition [6].

Because of its potential toxicity, hydrogen cyanamide is categorized by EPA into toxicity category I [7]. In European Union, according to the harmonized classification and labelling (Annex VI of CLP Regulation (EC) No. 1272/2008), this substance is: ‘toxic if swallowed, toxic in contact with skin, causes severe skin burns and eye damage, causes serious eye damage, suspected of causing cancer, suspected of damaging fertility and the unborn child, may cause damage to organs through prolonged or repeated exposure, harmful to aquatic life with long lasting effects and may cause an allergic skin reaction’ [8].

Hydrogen cyanamide exposure is primarily reported as unintentional in occupational settings, while reports of self-poisoning are less common [9]. The most common routes of exposure are dermal and inhalation. Hydrogen cyanamide is known to cause severe irritation: the most common symptoms are throat irritation, dyspnoea, vomiting, erythema and caustic burns of skin, mucous membranes and eyes [9, 10]. More severe skin reactions simulating Stevens–Johnson syndrome/erythema multiforme have also been reported [11]. Upon ingestion, clinical manifestations are more severe such as hypotension, hyperthermia, convulsions, coma and haematuria [9]; cases of fatalities have also been reported [12]. The exact mechanism underlining hydrogen cyanamide toxicity has not yet been established. Some animal experiments identified cyanide as a potential product of hydrogen cyanamide oxidation [13], suggesting the possible risk of cyanide poisoning, but further experiments failed to demonstrate that such conversion occurred in humans [14]. A proposed mechanism of action [15] is hydrogen cyanamide activation by a catalase and successive inhibition of the same enzyme causing uncoupling of oxidation and phosphorylation leading to blockage of adenosine nucleotide synthesis [9, 12].

Lastly, hydrogen cyanamide exhibits an inhibitory effect on aldehyde dehydrogenase (ALDH), carrying the risk to produce disulfiram–ethanol reactions (DER)-like when exposure occurs together with alcohol consumption [16]: a few cases have already been described in literature [17]. Interestingly, the same ability to inhibit ALDH is also possessed by another form of cyanamide, calcium cyanamide, a drug introduced in Canada, Europe and Japan in the sixties as chronic alcohol therapy [1].

The aim of this study is to evaluate all hydrogen cyanamide exposure cases referred to Pavia Poison Control Centre (PPCC) between January 2001 and December 2021, to describe how exposure patterns changed from its market withdrawal and to better characterize the clinical manifestations, with particular focus on signs and symptoms after concomitant hydrogen cyanamide use and alcohol consumption.

## Methods

This retrospective single-centre study was performed by the toxicology staff of Pavia Poison Control Centre. PPCC is an emergency service where clinical toxicologists advise, primarily medical physicians, on the management of intoxicated patients all over Italy. Therefore, it’s an observatory capable of intercepting toxicological problems even when rare and dispersed throughout the country. PPCC also collaborates with national and European institutions for surveillance activities in different toxicological areas [18,19]. Most of the consultation requests come from emergency departments and intensive care units of Italian hospitals, and for each patient, a digitalized medical record is filled out reporting demographic data, a narrative of the event, clinical picture at admission and throughout hospital stay, laboratory analysis, treatments and outcome. All the consultation requests for hydrogen cyanamide referred to PPCC from January 2001 to December 2021 were retrieved using specific keywords: Dormex and hydrogen cyanamide. All cases were anonymized, and the following data were analysed: age, sex, route of exposure (inhalation, ingestion, skin and/or eye contact), year and month of exposure, geographical location, use of personal protective equipment (PPE), intent of exposure (accidental, suicidal, professional), alcohol co-ingestion, signs, symptoms, treatments, and outcome. The clinical severity of each case was assessed by the clinical toxicologist who answered the call using the Poisoning Severity Score (PSS), including grades 0 (no symptoms or signs related to poisoning), 1 (mild, transient and spontaneously resolving symptoms), 2 (pronounced or prolonged symptoms), 3 (severe or life-threatening symptoms) and 4 (death) [20]. Cases with no causality between the suspected exposure and symptoms and preventive requests were excluded. The study was approved by our institutional review board. The study received local ethics committee approval (n. 2615 CEC). The data were analysed descriptively; quantitative data were presented as median (25th; 75th percentiles) while nominal data were presented as absolute values and proportions.

## Results

**Figure 1.**
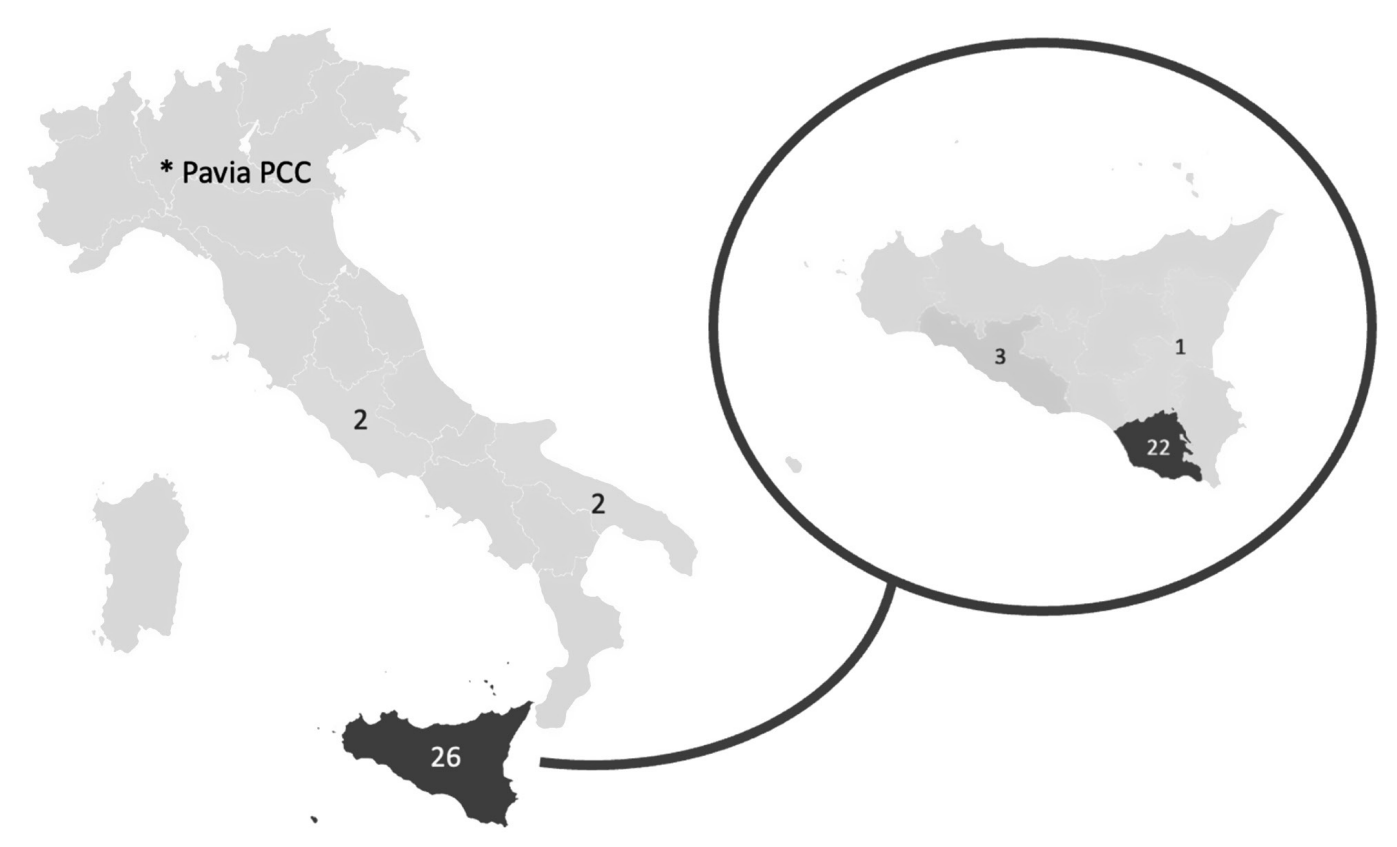
Geographical distribution of hydrogen cyanamide exposure cases.

Considering the analysed period, a total of 30 adult patients with hydrogen cyanamide exposure were included in the study. In all cases evaluated, Dormex was involved. Median case/year was 1,5 [1;2]. We observed a seasonality: the cases of Dormex exposures were generally reported from November to February (97% of cases) with a peak in January (14 cases). Considering geographical distribution, as shown in Figure 1, we saw a prevalence in the Sicily region (87% of cases) with only four cases (13%) reported outside of this region: two cases from Lazio and two cases from Apulia. Moreover, considering the province from which exposures were mostly reported, we noted that 73% (*n* = 22) of cases came from Ragusa (Southern Sicily). The median patient age was 50 [33,5;57,5] years. Twenty-nine patients (97%) were males. Thirteen patients (43%) had a concomitant alcohol ingestion. Patients’ demographic and exposures characteristics are summarized in Table 1 (patients without concomitant alcohol ingestion) and Table 2 (patients with concomitant alcohol ingestion).

**Table 1.** Summary of cases exposed to hydrogen cyanamide without concomitant alcohol consumption

Case number	Age, sex	Month, year	Route of exposure	PPE	PSS at presentation	Clinical manifestations
1	45, M	January, 2001	Ingestion (estimated 50 mL of pure product)	Un.	1	Nausea, vomiting, coma
2	50, M	January, 2006	Dermal	N	1	Cutaneous hyperaemia
3	50, M	February, 2006	Dermal-ocular	Y	1	Cutaneous hyperaemia of face, eyelid oedema, conjunctival hyperaemia
4	34, M	December, 2007	Inhalation (open space)	Un.	1	Weakness
5	63, M	December, 2008	Inhalation/dermal contact (closed space)	Y	1	Cutaneous hyperaemia of face, thorax and back, tachycardia
6	42, M	January, 2010	Dermal-ocular	Un.	2	II-degree burns on the back, eyelid oedema
7	30, M	January, 2011	Inhalation (open space)-dermal	Un.	1	Cutaneous hyperaemia, nausea
8	Un., M	February, 2011	Dermal	N	2	III-degree burn on fingers of the hand, oedema
9	65, M	December, 2011	Dermal	N	1	Cutaneous hyperaemia
10	74, M	January, 2014	Ingestion (15 ml estimated)	Un.	2	Nausea, vomiting, confusion, agitation, buccal ulcers
11	74, M	February, 2014	Ingestion (estimated 50 ml of pure product)	N	3	Agitation, confusion, coma, hemiplegia, pancreatitis
12	42, M	July, 2015	Ingestion (estimated 50 ml of pure product)	Un.	3	Nausea, vomiting, confusion, agitation, coma, hallucinations, hepatic necrosis, pancreatitis, corrosive esophagitis and gastritis
13	62, F	January, 2016	Inhalation (closed space)	N	1	Nausea, vomiting
14	41, M	January, 2019	Dermal	N	1	Cutaneous hyperaemia, Pharyngodynia
15	23, M	December, 2019	Inhalation-dermal	Un.	1	Headache, nausea, abdominal pain, cutaneous hyperaemia on the hand
16	24, M	January, 2020	Dermal	N	1	Cutaneous hyperaemia, oedema, burning sensation and pruritus on right upper limb and clavicular region
17	50, M	January, 2021	Dermal	Y	1	Cutaneous hyperaemia

Un.: unknown; Y: yes; N: no.

**Table 2.** Summary of cases exposed to hydrogen cyanamide with concomitant alcohol consumption

Case number	Age, sex	Month, year	Route of exposure	PPE	PSS at presentation	Clinical manifestations
18	26, M	January, 2001	Inhalation (open space)	Un.	1	Face and neck flushing, fatigue
19	20, M	February, 2001	Inhalation (open space)	Un	1	Face and body flushing, tachycardia
20	79, M	February, 2002	Inhalation	Un	1	Flushing, tachycardia, dyspnoea
21	52, M	January, 2007	Inhalation (open space)	Un.	2	Dyspnoea, body flushing
22	33, M	December, 2007	Inhalation	Y	1	Body flushing
23	50, M	February, 2008	Inhalation (open space)	Un.	1	Face and body flushing
24	53, M	January, 2009	Inhalation (open space)	Un.	1	Face flushing
25	Un., M	January, 2010	Inhalation (open space)	Un.	2	Pharyngodynia, dyspnoea, body flushing
26	Un., M	January, 2010	Inhalation (open space)	Un.	2	Pharyngodynia, dyspnoea, body flushing
27	63, M	November, 2011	Inhalation (closed space)	Un.	2	Dyspnoea, face and body flushing
28	28, M	December, 2013	Inhalation (open space)-dermal	Un.	2	Face and body flushing, wheals upper limbs and back, II-degree burn on thigh, drowsiness
29	51, M	December, 2018	Inhalation	Y	2	Dyspnoea, face flushing
30	45, M	December, 2021	Inhalation	Y	2	Dyspnoea, body flushing, hypotension

Un., unknown; Y, yes; N, no.

All cases are professional exposures: they all occurred at the workplace. In Italy, Dormex is primarily used in the cultivation of table grapes and cherries. Most of our patients worked in the context of table grape crops which is quite common in Sicily. Evaluating all consultation requests, 14 patients reported inhalation as the route of exposure, 6 dermal contact and 4 ingestion. In six cases, the contact routes were more than one (*n* = 2 dermal-ocular; *n* = 4 dermal-inhalation). As shown in Tables 1 and 2, accidental/unintentional exposure was the only reported event, with no cases of intentional self-poisoning. Exposure was mainly due to the habit of workers to keep a bin containing the product close to their body to easily nebulize it over the plants. That behaviour puts workers at risk of both product spillage with direct dermal contact and inhalation/dermal contact during nebulization, especially if used without the suggested PPE. In the four cases of ingestion, exposure was secondary to the transfer of the product into another container and successive drinking from that container. The median PSS at presentation was 1 [1;2].

**Figure 2.**
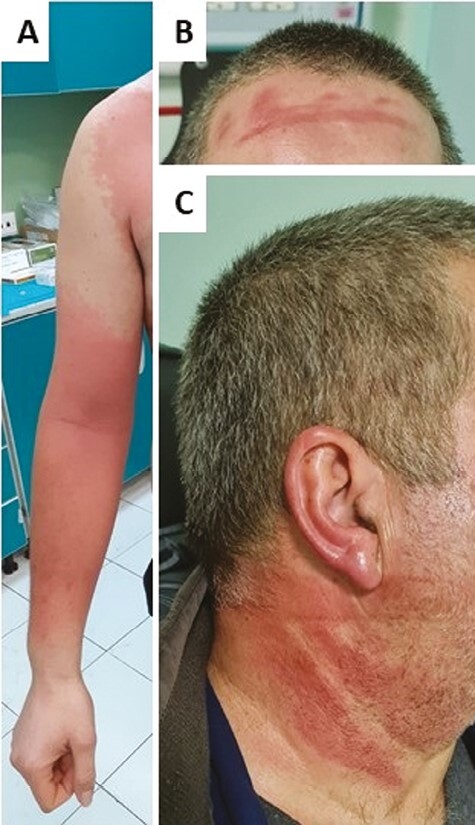
Cutaneous lesions related to hydrogen cyanamide direct contact. Please note the typical distribution of cutaneous toxic effects especially in not protected areas by PPE. For clinical details, refer to Table 1 (A for Case#16 and B, C for Case#17).

Clinical presentation depended on exposure mechanisms. If we considered dermal exposure only, the reported signs were mostly on contact site: cutaneous hyperaemia (n = 5), oedema (n = 2), III-degree burn (*n* = 1), pruritus (*n* = 1), burning sensation (*n* = 1) and pharyngodynia (*n* = 1) (Figure 2). Patient #8, was the only patient presenting with III-degree burn and had directly applied the product by hand for several hours without gloves. In the cases associated with solely inhalation, weakness (*n* = 1), nausea (*n* = 1) and vomiting (*n* = 1) were the reported clinical manifestations. Despite the dermal route being the only reported exposure route for patient #14, pharyngodynia was reported, suggesting a possible concomitant unnoticed inhalation. Hydrogen cyanamide exposures by more than one route were characterized by symptoms associated with both exposure routes. The two cases with both dermal and ocular contact were characterized by cutaneous hyperaemia (n = 1), cutaneous II-degree burn (*n* = 1), conjunctival hyperaemia (*n* = 1) and ocular oedema (*n* = 2); patient #6 also reported that the lesions had progressed over 2 days after the contact, while patient #3 was wearing a facial mask, leaving eyes and zygomatic bones uncovered. In cases of both dermal contact and inhalation, the reported symptoms were cutaneous hyperaemia (n = 3), headache (n = 1), nausea (n = 2), abdominal pain (n = 1) and tachycardia (n = 1).

We had four cases of ingestion, all accidental: they were associated with more severe PSS at arrival (PSS at presentation was 3 in 50% of cases). The reported symptoms were nausea (*n* = 2), vomiting (*n* = 3), oral caustic burns (*n* = 1), corrosive esophagitis and gastritis (*n* = 1), pancreatitis (*n* = 2), and hepatic necrosis (*n* = 1). Moreover, the following alterations of central nervous system were recorded: confusion/drowsiness (*n* = 3), agitation (*n* = 3), coma (*n* = 3), hallucinations (*n* = 1) and hemiparesis (*n* = 1). Cases #1, #11 and #12 developed severe clinical manifestations: progressive sensorium deterioration until coma with the need for intubation and intensive care admission. Case #11 was characterized by hemiplegia on day 3 and hospitalization in the ICU for 27 days until a complete recovery occurred. Case #12, instead, developed pancreatitis, hepatic necrosis, corrosive esophagitis and gastritis documented by esophagogastroduodenoscopy (EGDS) and, as complications, *ab ingestis* pneumonia and psychomotor agitations and hallucinations after extubation. Unfortunately, further attempts to follow up failed. Both cases #11 and #12 also had amylase and lipase increase. Specific laboratory findings regarding organ damage were available only for one patient (case # 11): lipase peak value was 472 UI/L (n.v. < 190), and amylase peak value was 210 UI/L (n.v. < 53). Case #10 was associated with milder symptoms, oral ulcers and EGDS negative for caustic lesions: the lesser amount of the product ingested could explain this clinical presentation. From a clinical point of view, none of our patients showed signs/symptoms of cyanide toxicity.

Considering hydrogen cyanamide exposure and alcohol co-ingestion, as shown in Table 2, patients reported the following symptoms: flushing (*n* = 13), dyspnoea (*n* = 7), pharyngodynia (*n* = 2), tachycardia (*n* = 2), confusion/drowsiness (*n* = 1), hypotension (*n* = 1), fatigue (*n* = 1), II-degree burn (*n* = 1), cutaneous wheals (*n* = 1). Case #28 was associated with the appearance of wheals on the back; direct contact in that skin area was not reported, and thus it could be secondary to a sensitization mechanism. Time elapsed from alcohol consumption and hydrogen cyanamide use was variable ranging from a few hours to a maximum of 24 hours (median time 5 hours [4;24]).

All patients were treated symptomatically since no specific therapy or antidote is known for hydrogen cyanamide. No competitive inhibitor of alcohol dehydrogenase (e.g. fomepizole) was administered to reduce the DER-like symptoms. In case of dermal/ocular contact was suggested rinsing with a saline sterile solution for at least 30 minutes and removal of contaminated clothes, followed by specific therapy according to burning degree (topical silver sulfatiadiazine, steroids, non-steroidal anti-inflammatory drugs, antibiotics and surgical revision of the lesion). When ingestion was the route of exposure, therapy consisted of anti-emetics, proton pump inhibitors, antibiotics and sedation in case of hallucination and agitation; intubation was necessary in three cases and EGDS was suggested in all cases, but feasible in two. When alcohol consumption was also present, hydration with saline solution was performed together with symptomatic therapy.

## Discussion

Poisoning by agricultural chemicals is a global public health problem long recognized by the WHO. Hydrogen cyanamide is part of this hazardous group but there are few data on exposure events. Despite Dormex being recalled from the Italian market in 2008, exposure events are still reported more than a decade later. From 2011, we saw a slight decrease in cases, but the cases/year ratio remains constant throughout the years. This indicates the ongoing use of the product possibly secondary to its illegal purchase. Monthly data show a seasonality, with peaks in December and January. This is consistent with previous Italian reports [5,10] and hydrogen cyanamide use: it’s generally applied 30–45 days before the expected bud break, in the colder months [21]. Interestingly, the geographic distribution shows a dramatic prevalence in Sicily, particularly in the Ragusa province: these are areas where winter chill is not enough to break bud dormancy in spring, a process that can be facilitated by hydrogen cyanamide [22].

In this study, hydrogen cyanamide exposures occurred only accidentally at the workplace, the trend in accordance with literature data [4,9]. After dermal-ocular contact, cutaneous manifestations ranged from mild hyperaemia to II-degree burn and we had only one case of III-degree burn. The exact mechanism by which hydrogen cyanamide can cause cutaneous manifestations has not been fully elucidated: both a toxic direct mechanism and an allergic/immune-mediated one were suggested [1, 11]. In our case series, lesions were located on the contact site, suggesting a direct toxic mechanism, but no allergic patch testing was done to better characterize them. Moreover, we had one case (#28) in which a potential sensitization mechanism could not be excluded. More severe generalized dermal manifestations, previously described [11, 23], were not found in our series.

This study shows that ingestion is associated with more severe symptoms, in accordance with other case series. Sharif et al. report that a more negative outcome correlates with ingestion of more than 100 mL of product and hallucinations, which occurred in 68% of patients [9]. Gamaluddin et al. reported coma and hallucinations in 83% and 42% of cases, respectively [2]. Unfortunately, neither of those studies correlate clinical manifestations to exposure route. Four fatalities have been reported after hydrogen cyanamide exposure. One case was an intentional ingestion of approximately 150 mL of it; the patient quickly becomes unconscious, deteriorating into cardiogenic shock 22 hours after admission [12]. Three other cases were reported by Gamaluddin et al. [2], but the route of exposure wasn’t specified.

Lastly, our study focused on alcohol–hydrogen cyanamide interaction. ALDH inhibition by cyanamide has been used in the past to facilitate alcohol abstinence in chronic alcoholics [24]. Hydrogen cyanamide has the same inhibitory effect possibly leading to DER like. DERs depend on acetaldehyde accumulation secondary to the inhibition of its conversion to acetate, following ethanol consumption [25]. Mild-to-moderate reactions are usually characterized by flushing, nausea, vomiting, dyspnoea, tachycardia and hypotension, while severe ones may cause life-threatening symptomatology [26, 27]: fatalities have been described [28, 29]. Given this possibility, Dormex safety sheet indicates to avoid alcohol consumption 24 hours before and 48 hours after its use. Data on DER-like manifestations after hydrogen cyanamide use are scarce in literature and not well characterized, reporting mild cases without severe manifestations and with spontaneous recovery [5, 17, 30]. The most common symptoms manifested by our patients were flushing (100%) and dyspnoea (60%). In some cases, flushing was misinterpreted as an allergic reaction and therefore treated with corticosteroids; the nature of symptoms was later clarified after PCC was contacted. All manifestations were of mild severity: moderate hypotension was reported in one case, and drowsiness in another one. All cases spontaneously resolved a few hours after alcohol consumption. Unfortunately, ethanol serum level was only available for one patient: however, alcohol consumption was known through history and the appearance of symptoms shortly after alcohol ingestion allowed us to identify a correlation. In 80% of these cases, the reported hydrogen cyanamide route of exposure was inhalation/dermal contact during normal usage of the product, and in 30% of cases, protective measures were used. The development of DER-like manifestations implies that even during normal use, the product may be absorbed enough to cause ALDH inhibition. Symptoms are usually short term and mild, but it’s not possible to exclude the potential development of more severe manifestations given the fact that in the past have been reported fatalities due to calcium cyanamide–alcohol interaction [31].

Symptomatic therapy, according to exposure route and manifestations, is for now the only option: no specific antidote exists. In case of ingestion, neurologic symptoms should be expected: coma may require intubation and ICU admission. Organ function should be monitored since evidence of organ damage has emerged. Moreover, EGDS should be considered to determine the extent of oesophageal and gastric lesions and to better plan follow-up.

This study has some limitations: its retrospective nature and the low number of patients, explained by the relative rarity of such events in a country in which its sale has been illegal since 2008. A previous case series [4] concluded that hydrogen cyanamide was associated with only modest toxicity compared to the one found in other studies [5]. The authors attributed the lower toxicity to the strict government and local regulations and to extensive workers’ training. Unfortunately, safe conditions in pesticide use are often unrealizable, especially in developing countries: the FAO/WHO Code of Conduct on Pesticide Management declares that ‘pesticides whose handling and application require the use of personal protective equipment that is uncomfortable, expensive or not readily available should be avoided’ [32]. PPCC gives a significant contribution in the surveillance of such events, also through collaboration with the INIH (National Centre for Chemicals, Cosmetics and Consumer Protection).

Our case series supports the potential severe poisoning arising from hydrogen cyanamide exposure. More efforts should be made to supervise illegal trade, since Dormex is still used in some parts of Italy. Further studies should be done to fill the knowledge gap on pathophysiology, therapy and outcome. Our work shows how too often workers disregard the warning not to consume alcohol: it’s important to educate them on the risk this behaviour is associated with. Due to winter chill reduction in temperate regions, artificial bud breaking with products such as hydrogen cyanamide will be an important strategy in the following years [22], leading to a potential rise in Dormex use. Ideal would be the identification of human-safer alternatives and implementation of preventive measures in those countries where its sale is legal.
